# Effect of lifestyle intervention for people with diabetes or prediabetes in real-world primary care: propensity score analysis

**DOI:** 10.1186/1471-2296-12-95

**Published:** 2011-09-13

**Authors:** Joris J Linmans, Mark G Spigt, Linda Deneer, Annelies EM Lucas, Marlies de Bakker, Luc G Gidding, Rik Linssen, J André Knottnerus

**Affiliations:** 1 Maastricht University, CAPHRI, Department of General Practice, P.O. Box 616, 6200 MD, Maastricht, The Netherlands; 2 Corporation of Primary Health Care Centres Eindhoven, Eindhoven, The Netherlands; 3 Maastricht University, Political Science Department, P.O. Box 616, 6200 MD, Maastricht, The Netherlands

## Abstract

**Background:**

Many lifestyle interventions for patients with prediabetes or type 2 diabetes mellitus (T2DM) have been investigated in randomised clinical trial settings. However, the translation of these programmes into primary care seems challenging and the prevalence of T2DM is increasing. Therefore, there is an urgent need for lifestyle programmes, developed and shown to be effective in real-world primary care. We evaluated a lifestyle programme, commissioned by the Dutch government, for patients with prediabetes or type 2 diabetes in primary care.

**Methods:**

We performed a retrospective comparative medical records analysis using propensity score matching. Patients with prediabetes or T2DM were selected from ten primary healthcare centres. Patients who received the lifestyle intervention (n = 186) were compared with a matched group of patients who received usual care (n = 2632). Data were extracted from the electronic primary care records. Propensity score matching was used to control for confounding by indication. Outcome measures were exercise level, BMI, HbA1c, fasting glucose, systolic and diastolic blood pressure, total cholesterol, HDL and LDL cholesterol and triglycerides and the follow-up period was one year.

**Results:**

There was no significant difference at follow-up in any outcome measure between either group. The reduction at one year follow-up of HbA1c and fasting glucose was positive in the intervention group compared with controls, although not statistically significant (-0.12%, *P *= 0.07 and -0.17 mmol/l, *P *= 0.08 respectively).

**Conclusions:**

The effects of the lifestyle programme in real-world primary care for patients with prediabetes or T2DM were small and not statistically significant. The attention of governments for lifestyle interventions is important, but from the available literature and the results of this study, it must be concluded that improving lifestyle in real-world primary care is still challenging.

## Background

Worldwide, an unhealthy lifestyle is one of the leading causes of preventable death [1]. Inactive lifestyle and obesity are highly associated with the risk of developing type 2 diabetes mellitus (T2DM) and the complications associated with this disease [2-5]. Many programmes to improve physical activity and dietary behaviour have been investigated. Randomised controlled trials have shown positive effects of combined lifestyle interventions on the development of T2DM in patients with impaired glucose tolerance [6]. In patients who already have T2DM, combined lifestyle interventions improved weight loss, diabetes control and cardiovascular risk factors [7]. However, the translation of these combined lifestyle interventions in community and primary care settings has been shown to be promising, yet challenging [8-12]. In addition, the effects of exercise-only programmes for patients with T2DM were small, even in randomised trial settings [13,14]. Furthermore, when investigated in primary care, lifestyle counselling interventions had marginal effects on cardiovascular risk [15], exercise-referral schemes showed a small increase in physical activity in adults [16] and group education for patients with T2DM had modest effects on weight loss and smoking cessation [17].

Based on the positive results observed in randomised trials and the change in emphasis that has taken place in primary care from a curative setting into a setting with an increasing focus on prevention, several countries have adopted large scale lifestyle intervention programmes for patients with prediabetes (impaired fasting glucose or impaired glucose tolerance) or T2DM in primary care. In the Netherlands, a nationwide programme aimed at improving physical activity and dietary behaviour in patients with prediabetes or T2DM in primary care was started in 2008, commissioned by the Dutch Ministry of Health, Welfare and Sports (VWS) [18]. As described above, lifestyle programmes in primary care are challenging. Therefore, information about their real-world effectiveness is crucial for healthcare providers, researchers and policy makers [19,20]. The aim of our study was to investigate the effectiveness of the Dutch lifestyle programme for patients with diabetes or prediabetes in real-world primary care setting, using regular medical registration to evaluate the observed effects.

## Methods

### Setting and study design

We conducted this study in The Eindhoven Corporation of Primary Health Care Centres (SGE), a corporation comprising ten primary healthcare centres providing care for approximately 60000 patients in the city of Eindhoven, the Netherlands. SGE continuously registers and stores data in the electronic primary care record, which can be used for research purposes. We investigated the differences between patients who participated in a nationwide lifestyle programme and patients who received usual care according to a diabetes management programme. Within this programme, patients have regular checks annually with their GP and quarterly (three times per year) with a diabetes practice nurse (DPN) and if necessary in between. Every patient receives lifestyle advice from the DPN. Patients go to a dietician for a consultation on nutritional advice when they are diagnosed with the disease and if they start insulin therapy. The DPN is trained in motivational interviewing.

We performed a retrospective comparative medical records analysis using propensity score matching to control for confounding by indication. During the intervention, neither healthcare providers nor patients were aware that this study would be conducted.

### The lifestyle programme

The Dutch Institute for Sports and Physical Activity (NISB) developed and implemented a lifestyle intervention programme for patients with (pre)diabetes in primary care: the BeweegKuur [18]. The development and implementation were funded by the Dutch Ministry of Health, Welfare and Sports. The programme was gradually implemented from 2008 in the Netherlands and in 2012 it should be accessible everywhere in the Netherlands. The programme was designed to be as pragmatic as possible, meaning that it should fit within the scope and possibilities of current usual care. To improve implementation, the NISB provided additional training for lifestyle coaches who supervised the programme. The intervention will be reimbursed by Dutch healthcare insurance companies if it shows to be effective.

The primary goal of the BeweegKuur was to increase physical activity and to improve dietary behaviour in primary care patients with (a high risk for developing) T2DM. Other goals were improvement of HbA1c, blood pressure, BodyMassIndex (BMI), cholesterol, smoking status, waist circumference and in the long-term prevention of T2DM and lowering the incidence of complications. Patients were eligible to participate if they did not meet the Dutch Standard for Healthy Physical Activity (exercising at least half an hour for five or more days a week) and were motivated to change their lifestyle. Patients could not participate if they had T2DM with three or more complications and/or with serious polypharmacy (more than five different drug categories) and/or with hypertension above 180/110 mmHg.

The intervention started with a referral to a lifestyle coach, usually a DPN or physiotherapist. After this referral, patients subsequently entered one of three different physical activity programmes and all patients had one consultation with a dietician. The lifestyle coach determined which of the three programmes was best suited for the patient, coordinated the programme and provided counselling for one year. Details of the intervention have been published elsewhere [18].

### Participants

SGE was one of the first organisations that participated in the BeweegKuur. Four out of ten SGE-centres had the possibility to participate in 2008. All patients with prediabetes or T2DM who met the criteria for the intervention could participate. Patients were invited during a regular diabetes check-up or a regular consultation with the GP.

**Figure 1:**
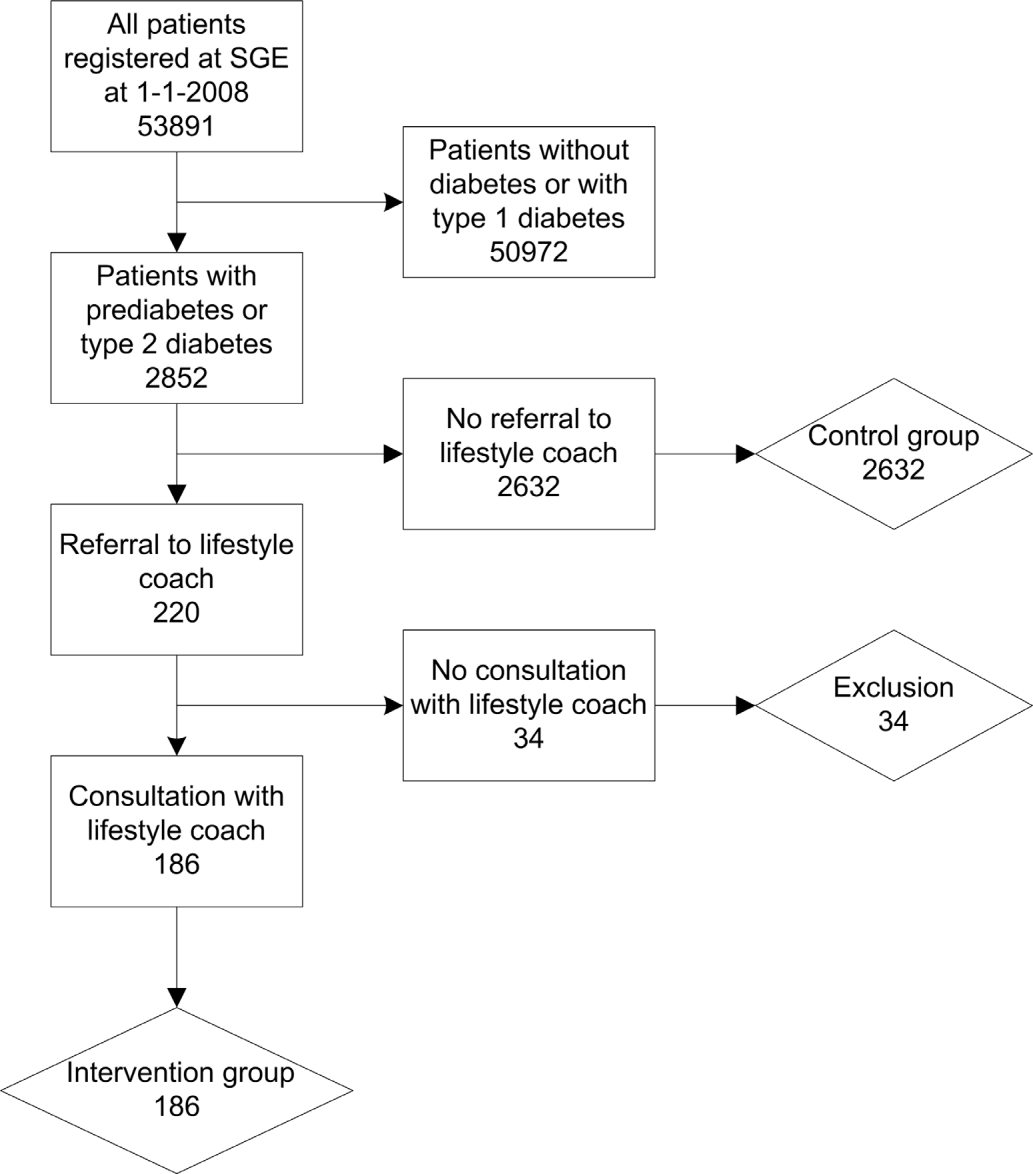
**Flowchart of study participants**.

For our analyses, we selected all patients with prediabetes (fasting plasma glucose 6.1-6.9 mmol/l) and all patients with T2DM (fasting plasma glucose > 6.9 mmol/l), registered with SGE on 1 January 2008, using the (ICPC) codes B85.01 (prediabetes) and T90.02 (T2DM) [21] (Figure 1). Subsequently, we examined all medical records of these patients to identify those who participated in the intervention at some point in 2008. We included the patients for analysis if they were referred to and had at least one consultation with the lifestyle coach. The control group consisted of all patients with prediabetes or T2DM from all ten centres of SGE who were not referred to the lifestyle coach.

The Medical Ethical Committee of the Maastricht University Medical Centre has approved this study.

### Outcome measures

As the intervention was designed to be part of usual care, all data of the outcome measures were extracted from the electronic primary care records. The outcome measures for diabetes patients were routinely registered during quarterly check-ups with the GP or DPN as part of the diabetes management programme and patients with prediabetes had similar check-ups. Therefore, data were collected for all patients with (pre)diabetes, regardless of their participation in the lifestyle programme. We extracted exercise level, BMI, HbA1c, fasting glucose, systolic and diastolic blood pressure, total cholesterol, HDL and LDL cholesterol and triglycerides. Exercise level was also monitored by the DPN during the regular quarterly check-ups. The level could be recorded as 1: sedentary lifestyle, 2: activities of daily living (e.g. grooming, dressing, eating) 3: healthy (exercising at least half an hour for five or more days a week) or 4: sports (more active than level 3). Weight and blood pressure were measured by the GP or DPN. Fasting glucose was measured in capillary blood and HbA1c, cholesterol and triglyceride in venous blood.

### Statistical Analyses

Since data collection was part of usual care, it was not possible to schedule follow-up measurements specific for this research. Therefore, we calculated means of all routinely recorded outcome measures for each patient one year before and after the date in 2008 on which the patient started the intervention. For the control group, we calculated means of the outcome variables using data of one year before and after 1 January 2008. In addition we investigated the effects solely using data of the last six months of the follow-up period of one year, to account for a possible weak or strong effect in the first six months. We only used those patients for analyses who had at least one measurement of the particular outcome measure in the year before and after the individual starting date.

A priori differences in patient characteristics between control group and intervention group may lead to biased estimates. In order to decrease this bias, we used propensity score matching techniques [22]. The propensity score of a person can be defined as the conditional probability of being exposed to a treatment given the person's covariates. For every person in the control group and the experimental group a propensity score was calculated. Using a logistic regression model we estimated the propensity of participating in the intervention for both intervention and control group based on a set of observed covariates. Matching covariates included: baseline score of specific outcome variables, age, gender, socio-economic status (based on postal codes) [23], marital status, smoking, COPD, asthma, cancer, cardiovascular disease, hypertension, disorder of lipid metabolism, cerebral ischemia, complaints of the locomotor system, neurologic disease, depression and mental illness. All these covariates were used to calculate the propensity score of all individuals. Baseline matching covariates were compared using independent t-test and chi-square test (SPSS 17.0). Propensity scores were calculated using Stata (version 10). All subjects in the control group were matched to subjects in the intervention group based on their propensity score, using a kernel matching algorithm [24]. We used the t-test to calculate the differences between both groups for all outcome variables. As physical activity was measured on an ordinal level and as we wanted to identify the changes in activity rather than categorise the level, we used the t-test for this variable as well.

## Results

In total 186 patients with prediabetes (n = 28) or T2DM (n = 158) participated in the BeweegKuur in 2008. The first patient started on March 1 and the last patient started on December 16, 2008. The matching covariates at study entry of both groups are shown in table 1. The imbalances between intervention group and control group at baseline as shown in table 1 endorse the need for matching. On average, patients in the intervention group were younger, were married more often and had cardiovascular diseases or COPD less often. The baseline outcome variables of study participants, as well as the unadjusted and adjusted mean effects of the lifestyle intervention are shown in tables 2 and 3.

**Table 1:** Covariates of the study participants at baseline used for matching.

Characteristics	Intervention group	Control group	*P*-value
n	186	2632	

Age (years)	62.93 ± 11.85	66.97 ± 12.56	0.00*

Sex male Sex female	44.1% 55.9%	47.5% 52.5%	0.37

Socio-economic status **	2.69 ± 0.79	2.75 ± 1.02	0.46

Married	59.7%	46.7%	0.00*

Diabetes mellitus type 2 **†**	84.9%	81.0%	0.18

Prediabetes **†**	15.1%	19.0%	0.18

Asthma **†**	5.9%	5.1%	0.64

Cancer **†**	12.9%	13.3%	0.88

Cardiovascular disease **†**	22.0%	32.0%	0.01*

Cerebral ischemia **†**	5.4%	9.0%	0.09

COPD **†**	1.1%	7.6%	0.00*

Depression **†**	9.1%	7.6%	0.45

Hypertension **†**	48.4%	50.6%	0.55

Locomotor system complaints **†**	43.5%	37.7%	0.11

Lipid metabolism disorder **†**	26.3%	21.7%	0.14

Neurologic disorder **†**	7.5%	8.8%	0.55

Psychic disorder **†**	10.2%	8.4%	0.40

Smoking **†**	5.9%	5.1%	0.64

Data are means ± SD or frequencies (%).

* Groups differed significantly (P < 0.05).

** Socio-economic status based on postal code of patient on a scale of 1-4 (1 = -2 to -1 SD compared to total population of SGE, 2 = -1 to 0 SD compared to total population, 3 = 0 to +1 SD compared to total population, 4 = +1 to +2 SD compared to total population) [23].

**† **Based on ICPC-codes.

**Table 2:** The effects of the lifestyle intervention compared with the control condition at one year follow-up.

Outcome variables	Means in Intervention group (SD)	Means in Control group (SD)	n Matched Intervention†	Adjusted effect of Intervention (95% CI)‡	Adjusted *P*-value
**BMI (kg/m2)**			152		

Baseline	30.36 (4.93)	29.54 (5.08)			

One year	30.33 (5.13)	29.63 (5.13)		0.04 (-0.87 to 0.94)	0.94

Exercise level (1-4)*			139		

Baseline	2.48 (0.60)	2.48 (0.71)			

One year	2.39 (0.73)	2.36 (0.71)		-0.01 (-0.18 to 0.17)	0.89

**Fasting glucose (mmol/l)**			184		

Baseline	7.21 (1.36)	7.28 (2.05)			

One year	6.98 (1.20)	7.18 (1.50)		-0.17 (-0.38 to 0.04)	0.08

**HbA1c (%)**			153		

Baseline	6.73 (0.92)	6.66 (0.97)			

One year	6.61 (0.75)	6.70 (0.87)		-0.12 (-0.30 to 0.06)	0.07

**Blood pressure**					

**Systolic (mmHg)**			176		

Baseline	138.98 (16.64)	143.68 (17.41)			

One year	138.59 (16.11)	142.90 (16.82)		-1.49 (-4.19 to 1.21)	0.25

**Diastolic (mmHg)**			175		

Baseline	79.88 (8.31)	79.39 (8.79)			

One year	79.35 (7.82)	79.17 (8.64)		-0.45 (-1.79 to 0.90)	0.48

**Total cholesterol (mmol/l)**			169		

Baseline	4.54 (0.94)	4.56 (1.00)			

One year	4.44 (1.00)	4.52 (0.98)		-0.12 (-0.26 to 0.22)	0.83

**LDL (mmol/l)**			169		

Baseline	2.80 (0.76)	2.85 (1.04)			

One year	2.67 (0.84)	2.73 (0.83)		0.01 (-0.19 to 0.21)	0.91

**HDL (mmol/l)**			169		

Baseline	1.16 (0.29)	1.16 (0.28)			

One year	1.18 (0.30)	1.17 (0.31)		0.03 (-0.05 to 0.07)	0.68

**Triglycerides (mmol/l)**			169		

Baseline	1.73 (0.98)	1.63 (0.95)			

One year	1.79 (1.01)	1.69 (1.19)		0.03 (-0.21 to 0.26)	0.76

† Number of patients from the intervention group matched with patients in the control group.

‡ The adjusted effect of the intervention after the matching procedure.

* Exercise level: 1 = sedentary lifestyle; 2 = activities of daily living; 3 = exercising at least half an hour for five or more days a week (NNGB); 4 = more active than NNGB.

**Table 3:** The effects of the lifestyle intervention compared with the control condition at months 7-12 of follow-up.

Outcome variables	Means in Intervention group (SD)	Means in Control group (SD)	n Matched Intervention†	Adjusted effect of Intervention (95% CI)‡	Adjusted *P*-value
**BMI (kg/m2)**			134		

Baseline	30.36 (4.93)	29.54 (5.08)			

7-12 months	30.29 (5.08)	29.51 (5.18)		0.27 (-0.60 to 1.14)	0.56

Exercise level (1-4)*			107		

Baseline	2.48 (0.60)	2.48 (0.71)			

7-12 months	2.22 (0.86)	2.27 (0.81)		-0.14 (-0.27 to 0.01)	0.12

**Fasting glucose (mmol/l)**			170		

Baseline	7.21 (1.36)	7.28 (2.05)			

7-12 months	7.07 (1.25)	7.17 (1.67)		0.08 (-0.11 to 0.26)	0.48

**HbA1c (%)**			94		

Baseline	6.73 (0.92)	6.66 (0.97)			

7-12 months	6.72 (0.83)	6.74 (0.90)		-0.10 (-0.23 to 0.03)	0.26

**Blood pressure**					

**Systolic (mmHg)**			166		

Baseline	138.98 (16.64)	143.68 (17.41)			

7-12 months	139.47 (16.55)	142.58 (17.47)		-1.49 (-4.04 to 1.05)	0.73

**Diastolic (mmHg)**			165		

Baseline	79.88 (8.31)	79.39 (8.79)			

7-12 months	78.71 (8.18)	78.53 (9.20)		-0.58 (-1.82 to 0.66)	0.40

**Total cholesterol (mmol/l)**			97		

Baseline	4.54 (0.94)	4.56 (1.00)			

7-12 months	4.56 (1.12)	4.52 (1.01)		-0.02 (-0.18 to 0.14)	0.87

**LDL (mmol/l)**			97		

Baseline	2.80 (0.76)	2.85 (1.04)			

7-12 months	2.74 (0.92)	2.72 (0.85)		0.10 (-0.04 to 0.23)	0.34

**HDL (mmol/l)**			97		

Baseline	1.16 (0.29)	1.16 (0.28)			

7-12 months	1.18 (0.28)	1.18 (0.29)		0.00 (-0.05 to 0.05)	0.96

**Triglycerides (mmol/l)**			97		

Baseline	1.73 (0.98)	1.63 (0.95)			

7-12 months	1.83 (0.95)	1.76 (1.48)		-0.20 (-0.37 to 0.03)	0.11

† Number of patients from the intervention group matched with patients in the control group. The numbers for this analysis are lower due to the fact that we did not have the necessary data for all participants in this shorter time frame.

‡ The adjusted effect of the intervention after the matching procedure.

* Exercise level: 1 = sedentary lifestyle; 2 = activities of daily living; 3 = exercising at least half an hour for five or more days a week (NNGB); 4 = more active than NNGB.

Overall, we found no relevant changes in both the intervention and control group. When using the propensity score matching, it showed that there were no statistically significant differences in effect between intervention group and control group during one year follow-up (table 2). There was a small positive, but not statistically significant effect of the intervention on HbA1c (-0.12%, CI = -0.30 to 0.06) compared with the control group (Table 2). Similar results were found for fasting glucose level (-0.17 mmol/l, CI = -0.38 to 0.04). There were no differences in exercise level, BMI, fasting glucose, blood pressure and cholesterol level between either group. Exercise level showed a small decreasing trend in both groups.

Similar results were found during the second half of the one-year follow-up period (Table 3). There was no significant difference in any outcome measure between either group. The exercise level in the intervention group was lower compared with the control group, although not statistically significant (p = 0.12). The positive trend of HbA1c observed during the first year was also seen during the last six months of the intervention, although with a higher p-value (p = 0.07 vs. p = 0.26). During the last six months of the follow-up period plasma triglyceride levels decreased, although not statistically significant (-0.20 mmol/l, CI = -0.37 to 0.03).

## Discussion

### Summary of results

In the Netherlands, a nationwide lifestyle programme for patients with prediabetes or T2DM was developed and implemented. We evaluated this lifestyle programme in real-world primary care. The effects of the programme compared with usual care during one year follow-up were small and not statistically significant or clinically relevant.

### Comparison with existing literature

Large randomised controlled trials of lifestyle programmes for patients with impaired glucose tolerance have shown significant positive effects on various outcome variables such as physical activity, HbA1c, weight, glucose, blood pressure and serum lipids after one year [25-27]. However, the results in our study follow a similar trend to the results in the Cochrane review by Thomas et al [14]. This review investigated the effects of exercise for type 2 diabetes mellitus and reported a decrease in HbA1c of 0.6% and a slight lowering of plasma triglycerides, but no other significant differences were found. Our results on the exercise level in the last six months of the intervention also resembles the findings of a review by Williams et al. [16] about exercise-referral schemes in primary care. These results showed that referral schemes had a small effect on physical activity, but that 17 patients needed to be referred for one to become moderately active. Causes of this high number needed to treat were poor rates of uptake and adherence to the exercise schemes. This might have happened in this lifestyle programme as well, despite being a programme designed and implemented to result in sustainable behaviour change [18]. Besides, our results underline the conclusion of the review and meta-analyses by Cardona-Morrell et al. [11], that showed that the translation of lifestyle interventions from randomised trials into routine practice has less effect on diabetes risk reduction.

Up to now, the translation of results achieved in randomised trials into routine clinical practice seems problematic [11]. Various factors could explain why the effectiveness of programmes seems to decrease when evaluated in real-world settings. Different aspects could add up to the positive effect of an intervention in a trial setting, as explained by Thorpe et al. [28] in their paper on differences between explanatory and pragmatic trials. For example, explanatory randomised clinical trials mainly use highly selected study participants, excluding patients with possible lower chances of a positive treatment effect. Besides, researchers and participants in trials follow strict and often intensive protocols, which could also lead to better adherence by the participants, but this might not be financially and practically feasible in real-world primary care.

### Strengths and limitations of the study

The primary aim of our study was to investigate the effectiveness of the intervention. Patients were analysed according to assignment to the intervention (referral to the programme and at least one consultation with the lifestyle coach) or usual care. We assumed that the effects after this consultation with the lifestyle coach were inherent to the intervention. In this study we had no data on treatment adherence after the first consultation. Therefore, we were unable to study whether poor treatment adherence could be the reason for the lack of effectiveness.

Due to the non-randomised design of this study we were not able to fully control for bias due to confounding. We used propensity score matching to overcome this problem. However, this does not take into account possible unmeasured confounding. Motivation to exercise for example was not measured routinely, so we could not control for this possible confounder. In addition, we were not able to control for information bias as a result of unblinded healthcare providers and patients. However, one would expect that patients in the intervention group were more motivated and that caregivers and patients were inclined to be biased towards a positive rather than a negative attitude towards the intervention. Consequently, this would rather lead to false positive results (bias from the null) than the absence of positive results as shown in our data. On the other hand, we cannot rule out bias to the null, for example by not referring patients who are very little motivated and who could benefit the most from the lifestyle intervention. In addition, we only used data from usual care electronic primary care records. However, this would only be considered real information bias if the quality or intensity of the registration depended on treatment exposure, but considering the strict registration criteria within the diabetes management programme, we assume this was not the case.

As regards the extrapolation of our findings beyond our study population, we have to consider that we evaluated the nationwide lifestyle programme in only a subset of healthcare centres, where we could be sure that medical registration was adequate. The narrow 95% confidence intervals indicate that we had sufficient statistical power. With a larger sample size some effects could become statistically significant, but it is unlikely that they will reach clinical relevance. The effects might be related to specific organisational aspects of health care centres. For example, it could be possible that the selected health care centres were already very active in lifestyle intervention, reducing the room for improvement. On the other hand, diabetes care in The Netherlands is highly structured according to a nationwide GP-guideline, so there should be no large differences in diabetes care.

## Conclusions

Because real-world investigations and interventions are necessary to really make a change in the diabetes epidemic, initiatives such as the BeweegKuur are very important in primary healthcare. The attention of the government for the increasing health and financial burden of diabetes is promising and nationwide lifestyle programmes are potentially relevant. On the other hand, considering the currently available literature and the results of our study, we should conclude that improving lifestyle in real-world primary care is still challenging. Qualitative research may be needed to find out how to improve the programme and to know what is important for patients and healthcare providers. Thorough process evaluations might reveal the barriers and facilitators for lifestyle intervention in diabetic patients in primary care.

## Abbreviations

T2DM: type 2 diabetes mellitus; VWS: Dutch Ministry of Health, Welfare and Sports; SGE: The Eindhoven Corporation of Primary Health Care Centres; NISB: The Dutch Institute for Sports and Physical Activity; BMI: BodyMassIndex; ICPC: International Classification of Primary Care; DPN: diabetes practice nurse.

## Competing interests

The authors declare that they have no competing interests.

## Authors' contributions

JJL, MGS and LD set up the design of the study. AEML and MDB generated the data. LGG, JJL and LD participated in the data extraction progress. RL, JJL, MGS and LD performed the statistical analyses and interpretation of the data. JJL, MGS and JAK drafted the manuscript. All authors critically reviewed and approved the final manuscript.

## Pre-publication history

The pre-publication history for this paper can be accessed here:

http://www.biomedcentral.com/1471-2296/12/95/prepub
